# Spatiotemporal trends in diabetes-related mortality by school district in the state of Michigan, United States

**DOI:** 10.4178/epih.e2021098

**Published:** 2021-11-09

**Authors:** Nurjannah Nurjannah, Kathleen M. Baker, Duduzile Phindi Mashinini

**Affiliations:** 1 Public Health Department, Medical School, Universitas Syiah Kuala, Banda Aceh, Indonesia; 2 Health Data Research, Analysis and Mapping (HDReAM) Center, Western Michigan University, Kalamazoo, MI, USA; 3 Department of Geography, Western Michigan University, Kalamazoo, MI, USA; 4 Interdisciplinary Health Sciences, Lecturer, College of Health & Human Services, Western Michigan University, Kalamazoo, MI, USA

**Keywords:** Diabetes mortality, Spatiotemporal analysis, Geographic information systems, Michigan, School district

## Abstract

**OBJECTIVES:**

This study examined the spatiotemporal epidemiological status of diabetes-related death in relation to school district boundaries in the state of Michigan, United States.

**METHODS:**

A retrospective observational study was conducted using death records spanning the years 2007–2014 in Michigan, with school districts as the geographic unit of analysis. Geocoding was performed for each death record. Cluster analysis used spatial autocorrelation with local Moran’s I, and spatiotemporal analysis used the Space Time Pattern Mining tool in ArcGIS Pro 2.1.

**RESULTS:**

The study revealed spatial clusters of high-high locations of diabetes-related mortality rate by school district in Michigan from 2007 to 2014. Spatiotemporal analysis showed grids with intensifying, consecutive, sporadic, and persistent hotspots of diabetes-related death in the Lansing, Royal Oak, Flint City, Berkley, Detroit City, East Lansing, South Lake, and Holt public school districts. These school districts should be prioritized for school-based diabetes prevention programs

**CONCLUSIONS:**

The study demonstrated the presence of various hotspots of diabetes-related deaths within the state of Michigan across the 8-year period of analysis. Understanding spatial and temporal hotspots could further improve our ability to evaluate diabetes burden across both time and space.

## INTRODUCTION

Type 2 diabetes is a preventable chronic disease. A structured lifestyle modification program for individuals with prediabetes can reduce their risk of developing type 2 diabetes by as much as 58% [1]. However, the Healthy People 2020 data indicated that in 2015, only 63.8% of adults aged 18 years or older engaged in leisure-time physical activity [2]. In addition, 36.7% of adolescents and 40.2% of adults stated that they consumed fruit less than once daily [3]. Physical activity and diet are among the risk factors associated with type 2 diabetes [4,5]. Therefore, preventive measures of diabetes should focus on modifying these factors.

Diabetes and obesity are influenced by American culture, which is characterized by the increasing consumption of higher-caloric food, sugar-sweetened drinks, and sedentary behavior [6]. Instead of focusing on adults, who may be less inclined to change these behaviors, there may be a better chance to influence behaviors in the younger population [6], and impact not only their current health, but also their future health [7,8]. Children spend most of their time at schools and supervised by teachers; therefore, schools provide an ideal setting for conducting lifestyle modifications, especially for the younger population [9]. Many studies have been conducted on diabetes prevention interventions in schools, suggesting that overall, school-based diabetes prevention programs offer promising outcomes for reducing risk factors for type 2 diabetes [10–14]. For instance, in study performed by the HEALTHY Study group, which utilized a school-based program to assess risk factors for diabetes among children, found that their school-based interventions significantly reduced children’s body mass index (BMI), which—as the authors pointed out—could reduce the risk of childhood-onset type 2 diabetes. Diabetes acquired during childhood is known to usher in a host of medical complications, some of which may not manifest until adulthood, including complications such as cardiovascular disease, which is the leading cause of death globally [10,15].

Seeing a grave need to address the risk of childhood diabetes, the Centers for Disease Control and Prevention (CDC) established a State Public Health Actions grant, which funds statewide programs that aim to prevent, manage, and reduce the risk factors associated with chronic diseases such as diabetes. The funded programs target childhood obesity, diabetes, heart disease, and stroke in 32 states, including Michigan [16]. Among other initiatives, the funding in Michigan is allocated to programs such as the Nutrition and Physical Activity Self-Assessment for Child Care, which seeks to increase consumption of healthy food and participation in physical activity among children from birth to the age of 5 years old [17]. Another Michigan-funded program aiming to promote healthy behaviors among children is led by the non-profit organization Blue Cross Blue Shield of Michigan, which oversees the school-based Building Healthy Communities (BHC) program. The BHC program aims to help children adopt healthy behaviors at a young age, targeting school children from kindergarten to 12th grade [18]. These programs all offer increasing access to healthy food and promote physical activity and seek to improve physical education through the school curriculum by providing training and equipment in a healthy and supportive environment [19].

While school-based diabetes prevention programs are being successfully implemented in Michigan, public health resources are not limitless; therefore, it is important that the programs are targeted in the areas corresponding to the most need. New ways to prioritize school-based diabetes prevention for targeted program implementation are critical for the efficient distribution of limited resources [20]. The aforementioned programs, which are currently being implemented in Michigan, may utilize information from studies that use diabetes-related mortality rate as a proxy for examining the diabetes burden by school district to prioritize areas of need, since data for diabetes risk are not widely available at the school district level. Diabetes mortality has been used as a measure to estimate the diabetes burden because it affects individuals’ functional capacities and quality of life, leading to significant premature mortality [21]. Previous studies have used diabetes death records to describe the diabetes burden at the school district level [22,23]. However, unlike previous studies, this study explored the diabetes burden by examining clusters of diabetes-related mortality rates and the trends exhibited by these clusters over an 8-year period across space and time, through a method known as spatiotemporal analysis.

Spatiotemporal analysis has been used to address a myriad of public health problems, such as traffic accidents among the elderly population in Seoul, Korea [24]. It has also been used to examine opioid-involved fatalities in Connecticut, United States [25]. Spatial analyses of diabetes-related mortality have been conducted in several studies in the United States [26,27] and China [28]. A study conducted in the United States by Dwyer-Lindgren et al. [26] analyzed spatial patterns in the mortality rate of various diseases at the county level, including diabetes, using death records from 1980 to 2014. Similarly, another study conducted in the United States by Kedir & Grigsby-Toussaint [27] used county-level data to examine clusters of diabetes-related mortality in 7 years of aggregated data (2003 to 2010); however, no temporal analysis was conducted in either study. While these studies have helped to inform our understanding of diabetes-related mortality and sound methodological techniques to examine it, more information is needed on the distribution of diabetes mortality across space and time, especially using emerging hotspot analysis, which enables the examination of different types of clusters such as intensifying, persistent, sporadic and new hot/cold spots to better understand trends over time at the school district level. Therefore, this study was designed to address this gap in analysis by assessing whether diabetes-related deaths occurred at random or clustered in terms of space and time in Michigan for the years spanning from 2007 to 2014. Through spatiotemporal analysis, the findings can better inform state government and local organizations about the diabetes burden by school district, thereby helping direct resources to high-need areas.

## MATERIALS AND METHODS

This retrospective observational study analyzed death records in Michigan from 2007 to 2014. All school districts in the state of Michigan, including elementary and unified school districts, were included in the study. Using the data from the United States Census TIGER/Line^®^ Shapefiles of the American Community Survey (ACS), 5-year estimates for the years 2009–2013, the study determined that there were 522 unified and 29 elementary school districts in Michigan during this time period [29].

### Data sources

For use in this study, death records were obtained from the Michigan Department of Health and Human Services through a data sharing agreement between the Kalamazoo Health and Community Services and the Health Data Research Analysis and Mapping (HDReAM) Center at Western Michigan University. All death records of people residing in Michigan between 2007 and 2014 were analyzed. To facilitate a comparison between deaths due to diabetes and all other causes, deaths were categorized as being related to diabetes or not. In this study, all deaths were defined as all deaths in the state of Michigan due to all causes during the designated time frame. Diabetes-related deaths referred to deaths with diabetes as the underlying or associated cause of death. In this study, diabetes was defined as the underlying cause as when diabetes directly led to death (part I of the death certificate) or when the associated cause stated that diabetes contributed to death but did not result in the underlying cause of death (part II of the death certificate) [30]. Both are coded E10 to E14 in the tenth revision of the International Classification of Disease [31]. The underlying and associated causes of deaths were used to ensure that all deaths related to diabetes were captured. Each deceased person who had diabetes codes as both the underlying and associated causes of death was analyzed only once, in the “underlying cause” category, to avoid duplication.

School district boundaries were retrieved from the United States Census TIGER/Line^®^ Shapefile [29] and the Michigan Center for Geographic Information website for both elementary and unified school districts [32]. They were combined for presentation and analysis, indicating a total of 551 school districts in Michigan [29].

To calculate the age-adjusted mortality rate, the population demographic estimates from the ACS 2009–2013 were used for the entire period of study to avoid introducing instability into the study results. These estimates are conducted by the United States Census Bureau and were also retrieved from the United States Census TIGER/Line^®^ Shapefiles [29] via the American data.census.gov website [33]. The age-adjusted mortality rate calculation used the number of individuals in the population that fell into 10-year age categories (with exceptions of groups for less than 5 years old, and 85 years and older).

### Geocoding (address matching)

Address matching was conducted for all deaths between 2007 and 2014 using ArcGIS^®^ version 10.3.1 [34]. As requested in the data sharing agreement, all analyses were conducted on an encrypted password-protected server to ensure confidentiality. The geocoding was conducted using a single field, in which the complete input address is stored in 1 field in the address table including house number, street, city, state, and postal code. There were 714,661 total deaths in Michigan from 2007 to 2014. The geocoding process was conducted by year with a total of 507,734 addresses matching (71.0%). For diabetes-related deaths, the study found 68,960 deaths matching a total of 58,209 addresses (84.4%). The remaining unmatched addresses were due to various errors such as incorrect spelling and abbreviated street names, as well as the spacing between words and numbers in addresses; as such, an analysis of these data points could not be performed. All matched addresses were spatially joined to school district boundaries for use in cluster analysis.

### Spatial and statistical analyses

To conduct a cluster analysis of diabetes-related mortality rates, diabetes-related deaths were calculated using the age-adjusted diabetes-related mortality rate by school district for each year from 2007 to 2014. Age-adjusted diabetes-related mortality rates were used to control for the effect of age distribution variations during the study period and among school districts. Mortality rates were directly standardized using the references of the United States population age distribution in the 2000 demographic census as recommended by the CDC and presented as deaths per 100,000 population [35,36].

Spatial autocorrelation analysis was conducted using the local Moran’s I statistic to locate clusters of mortality rates. First-order queen contiguity weights were used for all spatial analyses. Queen contiguity weights define neighbors as school district areas that share a boundary or a corner [37]. The p-value and z-score were obtained using the 999 permutations. The analysis was conducted using GeoDa [38], and the 8 temporal datasets from 2007 to 2014 were analyzed individually. The p-values lower than 0.05 and z-scores of 1.96 were considered to indicate statistical significance. The resulting maps were compared and visually examined to identify changes in clusters over time.

The Space Time Pattern Mining tool in ArcGIS Pro 2.1 was utilized to examine the spatiotemporal changes of diabetes-related deaths. This was used as an exploratory analysis tool for assessing spatial and temporal patterns of diabetes-related mortality to quantify the visual trends identified in the cluster analysis. The Space Time Pattern Mining toolbox includes the space time cube data structure and emerging hotspots analyses. The space time cube toolbox used point data of matched addresses. Each cube requires a standardized cell size (distance interval) input; in this study, a 3 km^2^ cell size was used because the smallest school district’s geographical area was 3.73 km^2^ [32]. Compared with other distance intervals of 1 km and 10 km (results not shown), the 3-km distance interval revealed more local variations while maintaining the non-zero bin count relatively high. Using a very small distance interval (1 km) resulted in many bins with zero-point counts affecting the cluster analysis. However, using a distance interval that was too large (10 km) concealed complex patterns in the cluster analysis that were evident with a distance interval of 3 km. The cube aggregates points into space-time bins [39]. Within each bin, the points were counted and the trend for bin values across time at each grid (location) was examined. As the cube used the Mann-Kendall statistic, which requires a minimum of 10 measurements [40], the study utilized 6-month bins, indicating deaths were counted in each 3 km^2^ grid for every 6 months from 2007 to 2014, resulting in 16 time series to avoid high zero bins in the analysis. The bins were initiated on the most recent year and built backwards. The time step alignment used in the space time cube development was “start time” to avoid temporal bias. By using “start time” in the time step alignment, the analysis only had a last time step temporal bias of 0.54%. Because the space time cube tool only aggregated point data, the rate was not calculated; therefore, it was not possible to conduct a direct comparison of the results between the space time analysis and the cluster analysis. To counter this, all death data was used and compared to the trend between deaths due to diabetes and all other causes. The same cell size and time series were used for all death data.

The Emerging hotspot tool was used to analyze identified cluster patterns over time. The tool analyzed temporal clustering using the space time cube results, which examined geospatial neighbors as well as the temporal bin neighbors for a feature using the Getis-Ord Gi* statistic [41]. The spatial relationship used in the emerging hotspot analysis was contiguity edges corners, which was used to be consistent with the cluster analysis of local Moran’s I. As the time step interval in the space time cube was 6 months, the 2 time-step intervals were selected for the neighborhood time step. This indicated that all bin counts within the neighborhood and all of their associated bins for the previous 2 time-step intervals (covering an 18-month period) were included in the analysis neighborhood. The results identified whether a bin had a new, consecutive, intensifying, persistent, diminishing, sporadic, historical, or oscillating value of either hot or cold spots [41]. The study of mortality did not allow negative values for an empty bin because there is no negative number for deaths. The lowest value was zero, indicating no mortality. All the significant hotspots from diabetes-related deaths were overlaid onto the hotspots from all deaths to examine which grids (locations) had a higher number of diabetes deaths relative to other grids.

According to Esri, the manufacturer of ArcGIS^®^, new hotspots are referred to as locations that are statistically significant hotspots for the most recent time step interval and have never been a statistically significant hotspot before [41]. Consecutive hotspots are locations with a single uninterrupted run of statistically significant hotspots at recent time step intervals, and comprised of less than 90% of all intervals [41]. Persistent hotspots are locations that are statistically significant hotspots in at least 90% of the time step intervals, with no trend up or down. Intensifying hotspots are defined as locations that have been statistically significant in at least 90% of the space-time period with an increasing intensity of clustering [41]. The opposite of intensifying hotspots is diminishing hotspots, where locations are statistically significant in at least 90% of the space-time period but the intensity is decreasing over time. Next are sporadic hotspots, where locations are statistically significant hotspots in a certain time period but do not constitute a significant cluster in others, and go back and forth in a sporadic fashion. Finally, historical hotspots are locations where statistically significant hotspots are present in at least 90% of the time step intervals, but not in the most recent time step intervals [41]. Because the aim of this study was to examine trends over time to target school-based diabetes prevention programs, the focus was only on new and intensifying hotspots of diabetes-related deaths compared to the hotspots of all-cause deaths to examine the consistency in trends over time between the 2 datasets. The resulting grids were layered with school district boundaries to determine which school districts should be targeted for school-based diabetes prevention programs.

### Ethics statement

The study was approved by the Western Michigan University Institutional Review Board (IRB Project No.: 14-09-27).

## RESULTS

**Figure 1:**
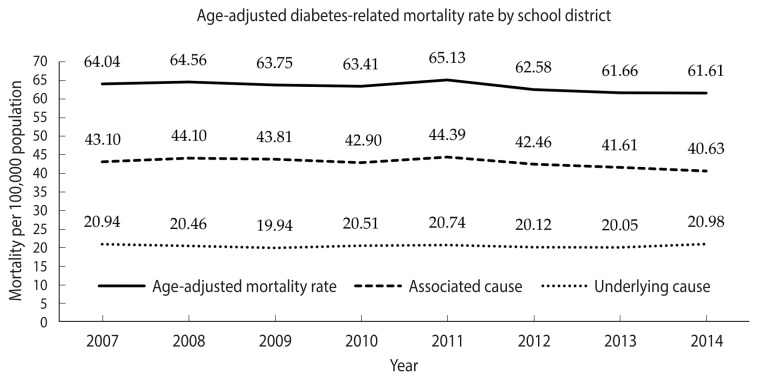
Age-adjusted mortality of diabetes at the school district level from 2007 to 2014 in Michigan.

During 2007–2014 (Figure 1), the age-adjusted mortality rates with diabetes as the associated cause slightly declined among school districts from 43.1 to 40.6 per 100,000 population, and those of diabetes as the underlying cause remained steady at approximately 20 per 100,000 population, resulting in a slight decline of diabetes-related mortality rates from 64.0 to 61.6 per 100,000 population. Overall, the rate of diabetes as the associated cause of death among school districts was 2 times higher than that for diabetes as the underlying cause of death.

The mortality data from 2007 to 2014 identified clustering of diabetes mortality by school district using the local Moran’s I analysis. Spatial autocorrelation was noted for diabetes-related mortality rate in all years studied, with Moran’s I values ranging from 0.02 in 2009 to 0.19 in 2014, all of which were statistically significant (p<0.01 for each year).

The local Moran’s I analysis identified spatial cluster and spatial outlier locations [38]. The spatial clusters were referred to as high-high or low-low clusters, where school districts with high diabetes-related mortality rates were surrounded by school districts with similar high rates, and school districts with low rates were surrounded by school districts with rates that were similarly low [38]. Spatial outliers were defined as high-low locations, where high rates were surrounded by low rates, or low-high locations, where low rates were surrounded by high rates [38]. As the aim of the study was to provide information for targeting school-based diabetes prevention programs, the results focused on high-high locations.

**Figure 2:**
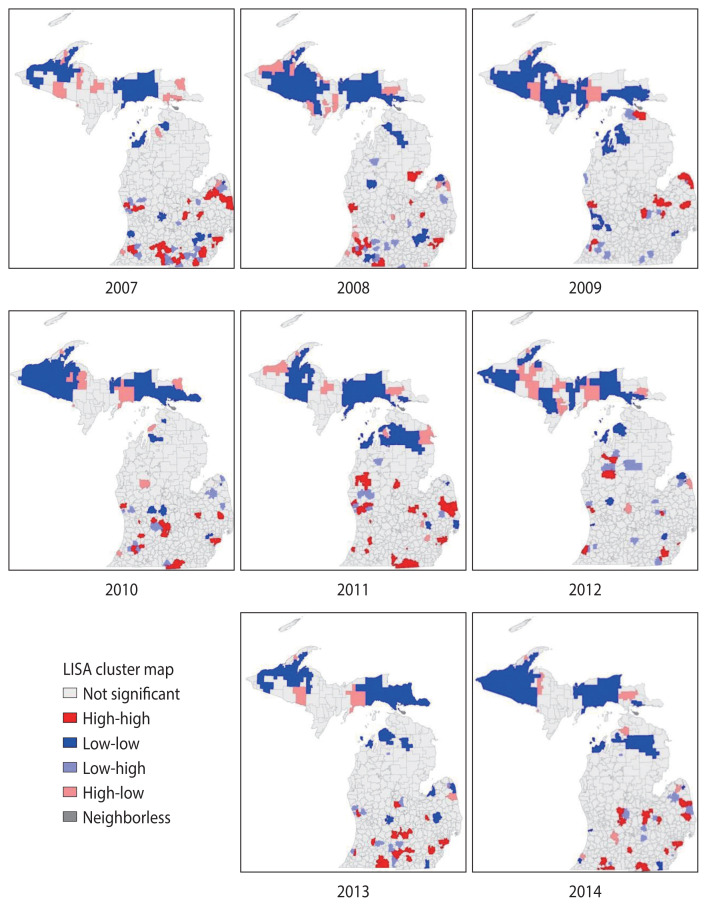
Eight-year trends in the age-adjusted diabetes-related mortality rate for Michigan by school district using cluster analysis of local Moran’s I with a significance filter (p<0.05). LISA, local indicator of spatial association.

The series of maps displayed in Figure 2, created for the years 2007–2014 and analyzed in the cluster analysis, demonstrated spatial clusters of high-high locations (in red on the map) of diabetes-related mortality rate by school district in the West and East Central, Southwest, and Southeastern Lower Peninsula of Michigan. No high-high clusters were identified in the Upper Peninsula of Michigan in all years of the study period. The number of school districts in the high-high clusters decreased from 40 school districts in 2007 to 21 school districts in 2014. Not all school districts were consistently in high-high clusters from 2007 to 2014. A visual examination indicated that there were temporal changes of high-high clusters school districts from 2007 to 2014. For example, the Flint City and Beecher Community school districts were consistently in high-high clusters without any single interruption within the 8 years of the study, while the Detroit City, Westwood Community, and Kalamazoo public school districts were in high-high clusters with at least 2 years of interruption. To be able to examine further the changes in diabetes mortality over time, the Space Time Pattern Mining tool in ArcGIS Pro was used.

### Spatiotemporal results

The results from the Space Time Pattern Mining analysis included all death data across the 8-year study period for school districts in Michigan. The space time cube results showed that the overall data trend was similar for both data for all-cause deaths and diabetes-related deaths, indicating a decreasing trend over time with negative cluster statistic values of −1.39 and −0.99, respectively. However, both p-values indicated that the results were not statistically significant, 0.16 and 0.32, respectively. Although the overall trends were not statistically significant, the trend for each grid indicated that it may be significant and was further examined in the emerging hotspot analysis.

**Figure 3:**
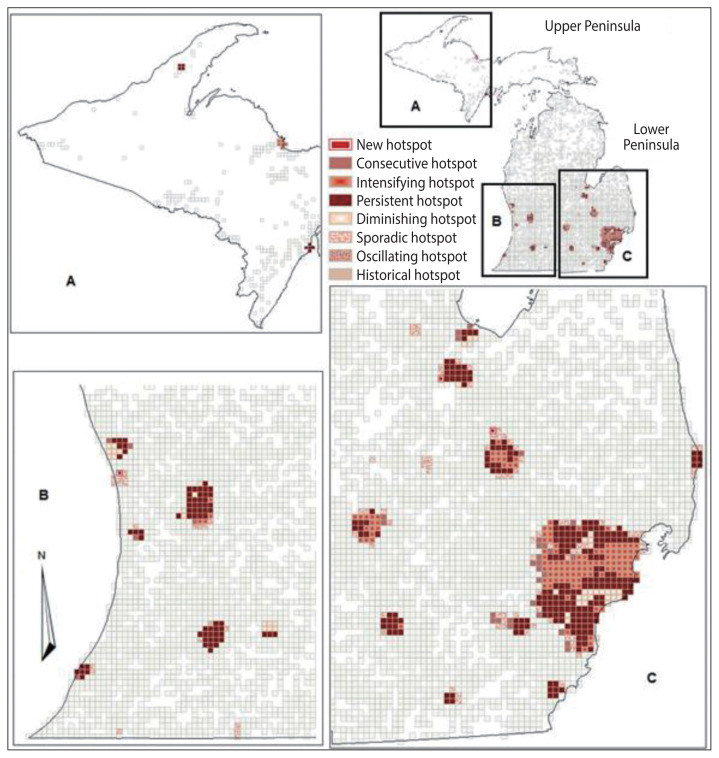
Trends in hotspot results of diabetes-related deaths in Michigan school districts, 2007–2014 (A) Western Upper Peninsula, (B) Southwest Lower Peninsula, and (C) Southeast Lower Peninsula.

**Figure 4:**
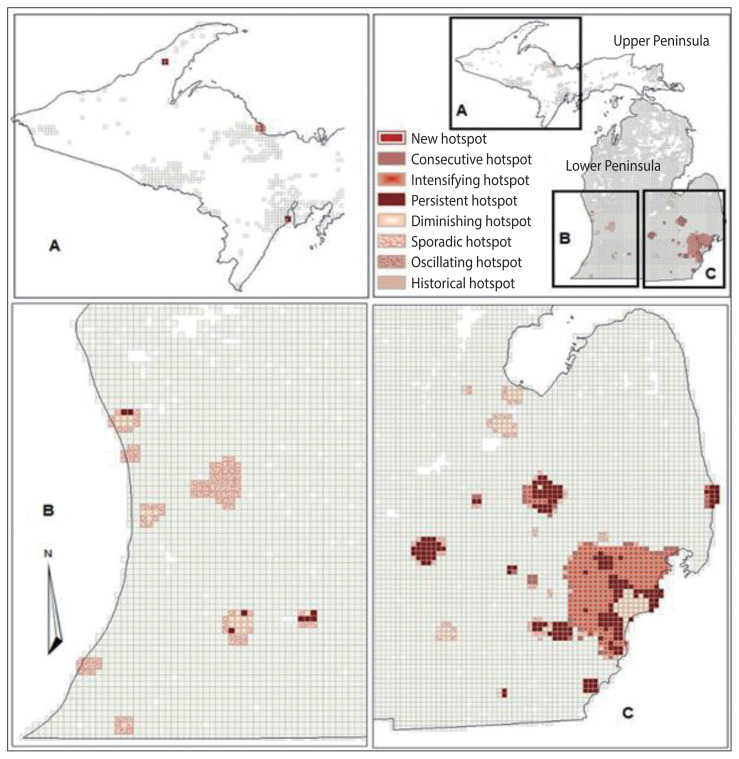
Trends in hotspot results of all deaths in Michigan school districts, 2007–2014 (A) Western Upper Peninsula, (B) Southwest Lower Peninsula, and (C) Southeast Lower Peninsula.

The results from the emerging hotspot analysis did not detect any cold spots (Figures 3 and 4). This is consistent with the mortality count dataset used, which had a high number of grids with zero values, indicating no mortality during the study period. The non-zero bins were lower in diabetes-related deaths (23.28%) than in all-cause deaths (46.32%), because the proportion of diabetes-related deaths was only 13.58% of total matched-addresses of all-cause deaths included in the study (Table 1). Both the datasets for all-cause deaths and diabetes-related deaths showed that the hotspots were mostly located in the West, East Central, Southwest, and Southeastern region of the Lower Peninsula school districts of Michigan, which is consistent with the result from the cluster analysis using local Moran’s I, and a few in the East Upper Peninsula (Figures 3 and 4). Table 1 shows that the results identified 7 types of hotspots: new, consecutive, intensifying, persistent, diminishing, sporadic, and historical hotspots with persistent and intensifying hotspots as the 2 highest hotspots for both datasets.

**Figure 5:**
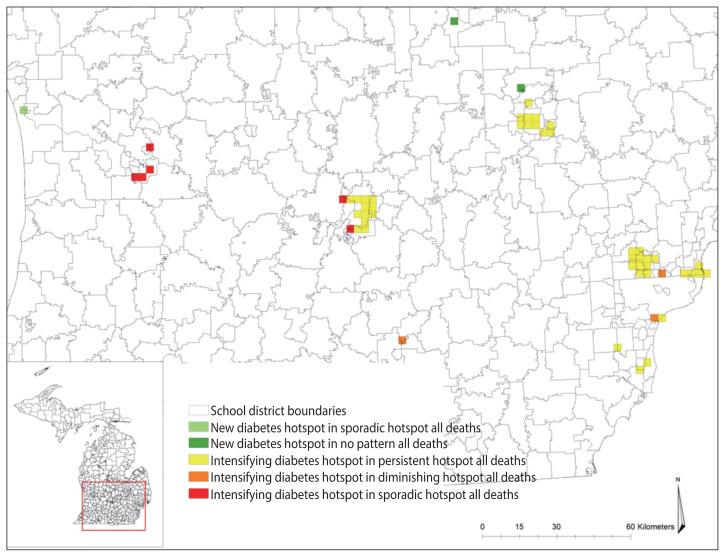
The comparison of new and intensifying diabetes hotspots in sporadic, persisten, diminishing, and no pattern hotspots for all deaths ini Michigan school districts, 2007–2014.

After layering the grids of diabetes-related deaths over the grids of all deaths, the results showed that there were school district locations in diabetes-related deaths with trends that were not consistent with the trend in overall deaths (Table 2), which is displayed in Figure 5.

The map in Figure 5 shows the differences in the hotspots between diabetes-related deaths and all-cause deaths in Michigan school districts. These hotspots are considered as the grids of concern where diabetes-related deaths increased while all-cause deaths did not. For example, the grids in green indicated that the overall deaths were either sporadic or had no cluster pattern existing over time, but diabetes-related deaths were statistically significant, increasing in 2014, making those locations new hotspots for diabetes-related deaths. Similarly, the grids in yellow indicated that all-cause deaths were in persistent, sporadic, or diminishing hotspots, but diabetes-related clusters were increasing over time.

The grids of concern were found in Genesee, Ingham, Jackson, Kent, Macomb, Saginaw, Oakland, Ottawa, and Wayne Counties. To determine which school districts to target for prevention efforts, the concerned grids were layered over the school district boundaries. The layering identified that the Lansing, Royal Oak, Flint City, Berkley, Detroit City, East Lansing, South Lake and Holt public school districts had at least 4 or more grids intersecting with the school district boundaries. These are the school districts that may be prioritized for school-based diabetes prevention program efforts.

## DISCUSSION

Age-adjusted diabetes-related mortality rates slightly declined among Michigan school districts between 2007 and 2014 from 64.0 to 61.6 per 100,000 population. This slight decline was consistent with 2014 death reports released by the CDC indicating a significant decrease in diabetes mortality rates in Michigan [42]. Although the report only included diabetes as the underlying cause of death, this may be related to the decrease in all diabetes deaths including diabetes as the associated cause. However, regardless of the year studied, diabetes-related mortality rates varied greatly by school district in this study, with similar statistically significant geographic cluster patterns in every year from 2007 to 2014 as determined by study results. That is, individuals residing in neighboring school districts were more similar in terms of their likelihood to die due to diabetes than individuals living farther away. This characteristic is consistent with Tobler [43] first law of geography that neighboring locations are likely to have similar characteristics. This shows that diabetes-related mortality is not a random phenomenon. This analysis is particularly useful to identify which communities, school districts have remarkably high rates or low rates of diabetes-related mortality in the context of their neighbors; this method has been used in a variety of public health research studies. For example, these analyses were used for determining spatial clusters of diabetes prevalence and its associated risk factors [44], clusters of BMI among adults with diabetes [45], and geographic disparities in the diabetes-related mortality rate across counties in the United States [27]. This information can be directly used for targeting communities with diabetes prevention and intervention efforts.

Although the local Moran’s I was used to analyze spatial patterns of diabetes-related mortality that existed in the data, showing high-high clusters in the East and West Central, Southeast, and Southwest Lower Peninsula school districts for yearly assessments from 2007 to 2014, the analysis did not indicate whether the mortality rates increased or decreased in those clusters. Thus, it is important to examine both spatial and temporal patterns in diabetes-related deaths in Michigan school districts by examining temporal changes in the 8-year data using the Space Time Pattern Mining tool that take advantage of not only the spatial aspect of the data, but also the temporal aspect of the data.

The emerging hotspot analysis produced new, consecutive, intensifying, persistent, diminishing, sporadic, and historical hotspots using the 2007–2014 dataset, although the space time cube did not contain significant trend results, presumably due to the length of time interval used in the analysis. If data were available for 10 or more years, a temporal bin size of 1 year could be utilized, which may reduce the time interval and improve the results. These hotspot locations are proximal to the location identified in the yearly local Moran’s I result in Figure 2, and may suggest quite similar hotspots with the exception of a few locations in school districts in the Upper Peninsula of Michigan (Figures 3 and 4). The hotspots may not exactly overlap because of the different measures and geographical level of analysis used between the local Moran’s I and emerging hotspot analyses. In the local Moran’s I analysis, the geographical level of the analysis of school district and mortality rate were used, while in the emerging hotspot analysis, cubes of 3 km^2^ and the mortality count were used; this resulted in the modifiable areal unit problem (MAUP) phenomenon, introduced by Openshaw [46], in which different results are obtained from the analysis of the same data that are aggregated into different groups of geographic levels. However, Manley et al. [47] established that MAUP is not a problem; instead, it is a resource. Data aggregated at different geographic levels can help us identify processes working at different levels [47]. It is obvious that it is not possible to define an ideal single geographic level that captures all the processes for all variables, especially for disease distribution. This study used the geographic level of the school district for local Moran’s I and identified high-high clusters of diabetes-related mortality rates, mostly in school districts located in highly dense, urbanized areas (no clusters were found in the Upper Peninsula of Michigan). When using the smaller geographic level (3 km^2^) instead of the school district, the study identified the spatiotemporal change of a few areas in the Upper Peninsula (Figures 3 and 4). This is a strategy that a study by Matisziw et al. [48] suggested is useful, stating that downscaling of the spatial structure of polygonal units could offer valuable information on the spatial pattern of disease.

From all the hotspots identified in the emerging hotspot analysis, the focus was given to intensifying and new hotspots because these areas indicated that an increased number of diabetes-related deaths occurred from 2007 to 2014. After comparing the hotspots between all-cause deaths and diabetes-related deaths, grids were specified that were in intensifying and new diabetes hotspots, while all-cause deaths showed persistent or diminishing hotspots. By intersecting the school district boundaries and the grids of intensifying and new hotspots, the study revealed the school districts that had an increasing number of diabetes-related deaths compared to others. These school districts included Lansing, East Lansing, and Holt school districts, which are located in between Ingham, Eaton and Clinton Counties; the Flint City school district in Genesee County, as well as the Berkley, Detroit City, Royal Oak, and South Lake school districts in Oakland, Macomb, and Wayne Counties. The results of this study indicate that public health officials may want to prioritize these school districts for targeted school-based diabetes prevention programs.

The methodology used in this study was able to examine the spatial and temporal clustering of diabetes-related mortality in the state of Michigan. This analysis added upon previous work by investigating multiple years of diabetes-related mortality data at the county level [26,27], while combining spatial and temporal components, rather than studying them separately. The results provide information about the spatiotemporal trends of diabetes-related deaths that can be used to make better decisions in implementing diabetes prevention programs as well as to evaluate the effectiveness of the implemented programs over time.

The limitations of this study include the inability of the Space Time Pattern Mining tool to easily support the current results as it requires a longer time period than was available in the current dataset to be set in an annual bin size. Additionally, the geocoding process may exclude unmatched addresses, lowering the number of deaths included in the analysis. The study also did not take into account other potential confounders in determining variation in the diabetes mortality rate by school district, such as socioeconomic status, healthcare facilities, and educational level. Finally, the migration of individuals in and out of school districts was also not accounted for in this study.

The combined spatial and temporal analyses used in this study demonstrated a novel method for using available death record data to target school-based diabetes prevention programs at the statewide level, particularly in Michigan. Future work should explore the application of these techniques to a dataset with a longer time span. In addition, the methods used in this study could be adapted to other public health problems and target populations by using different secondary health databases.

In conclusion, this study demonstrated the presence of diabetes-related mortality hotspots within the state of Michigan at the school district level. The results indicated that diabetes-related mortality is not a continuous feature statewide, but rather varies across both space and time. Furthermore, the patterns of diabetes-related mortality can vary in quite different ways from that of the full data set encompassing all-cause deaths in the state of Michigan. Understanding spatial and temporal hotspots could further improve our ability to design future diabetes prevention programs that are targeted to high-risk communities to ensure that limited public health resources are allocated where they are needed most, and may even be utilized to evaluate the effectiveness of programs that have already been implemented.

## Figures and Tables

**Table 1:** Emerging hotspot analysis results of all deaths and diabetes-related deaths in Michigan school districts, 2007–2014

Emerging hotspot analysis	All deaths (%)	Diabetes-related deaths (%)
New hotspots	1 (0.0)	3 (0.0)
Consecutive hotspots	37 (0.3)	34 (0.5)
Intensifying hotspots	229 (2.0)	131 (1.8)
Persistent hotspots	201 (1.8)	315 (4.4)
Diminishing hotspots	91 (0.8)	47 (0.7)
Sporadic hotspots	193 (1.7)	101 (1.4)
Oscillating hotspots	0 (0.0)	0 (0.0)
Historical hotspots	0 (0.0)	2 (0.0)
No pattern detected	10,426 (93.3)	6,529 (91.2)

**Table 2:** Comparison of hotspots in diabetes-related and overall deaths in Michigan school districts, 2007–2014

All causes of death hotspots	Diabetes-related deaths hotspots							
	New	Consecutive	Intensifying	Persistent	Diminishing	Sporadic	Historical	No pattern
New	0	0	0	0	0	0	0	1
Consecutive	0	1	0	1	0	4	0	29
Intensifying	0	13	80	84	6	25	0	19
Persistent	0	8	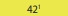	107	9	24	0	8
Diminishing	0	3	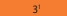	58	21	3	1	2
Sporadic	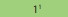	7	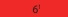	61	11	30	1	71
Historical	0	0	0	0	0	0	0	0
No pattern	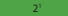	2	0	4	0	15	0	6,399
Total	3	34	131	315	47	101	2	6,529

1 Cells highlighted are the locations of concern for diabetes deaths that match the color in Figure 5.

## References

[1] Centers for Disease Control and Prevention (2016). Chronic disease prevention and health promotion.

[2] Office of Disease Prevention and Health Prevention (2016). Physical activity.

[3] Centers for Disease Control and Prevention (2017). Nutrition, physical activity, and obesity: data, trends and maps.

[4] Gillett M, Royle P, Snaith A, Scotland G, Poobalan A, Imamura M (2012). Modifiable risk factors for type 2 diabetes mellitus. Non-pharmacological interventions to reduce the risk of diabetes in people with impaired glucose regulation: a systematic review and economic evaluation.

[5] Murea M, Ma L, Freedman BI (2012). Genetic and environmental factors associated with type 2 diabetes and diabetic vascular complications. Rev Diabet Stud.

[6] Gregg EW (2010). Are children the future of type 2 diabetes prevention?. N Engl J Med.

[7] Robert Wood Johnson Foundation (2011). Early childhood experiences: laying the foundation for health across a lifetime.

[8] Lawrence RS, Gootman JA, Sim LJ (2009). Adolescent health services: missing opportunities.

[9] Peterson KE, Fox MK (2007). Addressing the epidemic of childhood obesity through school-based interventions: what has been done and where do we go from here?. J Law Med Ethics.

[10] Foster GD, Linder B, Baranowski T, Cooper DM, Goldberg L, HEALTHY Study Group (2010). A school-based intervention for diabetes risk reduction. N Engl J Med.

[11] Bani Salameh A, Al-Sheyab N, El-Hneiti M, Shaheen A, Williams LM, Gallagher R (2017). Effectiveness of a 12-week school-based educational preventive programme on weight and fasting blood glucose in “at-risk” adolescents of type 2 diabetes mellitus: randomized controlled trial. Int J Nurs Pract.

[12] Gortmaker SL, Peterson K, Wiecha J, Sobol AM, Dixit S, Fox MK (1999). Reducing obesity via a school-based interdisciplinary intervention among youth: Planet Health. Arch Pediatr Adolesc Med.

[13] Treviño RP, Hernandez AE, Yin Z, Garcia OA, Hernandez I (2005). Effect of the bienestar health program on physical fitness in low-income Mexican American children. Hisp J Behav Sci.

[14] Shaw-Perry M, Horner C, Treviño RP, Sosa ET, Hernandez I, Bhardwaj A (2007). NEEMA: a school-based diabetes risk prevention program designed for African-American children. J Natl Med Assoc.

[15] World Health Organization (2021). Cardiovascular diseases (CVDs).

[16] Centers for Disease Control and Prevention State Public Health Actions (1305) 2013–2018.

[17] Gilmore L Michigan early child care: improving nutrition and physical activity standards.

[18] O’Brien M (2016). Blue Cross Blue Shield of Michigan. Nearly 150 schools statewide to join innovative health and wellness-based building healthy communities program.

[19] Blue Cross Blue Shield Michigan (2017). Building healthy communities program elementary school program middle school step up for school program wellness program.

[20] National Conference of State Legislatures (2016). Diabetes health coverage: state laws and programs.

[21] Khan MA, Hashim MJ, King JK, Govender RD, Mustafa H, Al Kaabi J (2020). Epidemiology of type 2 diabetes - global burden of disease and forecasted trends. J Epidemiol Glob Health.

[22] Nurjannah N, Baker KM (2020). Using GIS and death records to inform statewide school-based diabetes prevention interventions in Michigan. J Public Health Res.

[23] Nurjannah N, Curtis AB, Baker KM, Paul R (2021). Mapping diabetes burden by school-district for school-based diabetes prevention interventions in selected cities in Michigan, USA. Geospat Health.

[24] Kang Y, Cho N, Son S (2018). Spatiotemporal characteristics of elderly population’s traffic accidents in Seoul using space-time cube and space-time kernel density estimation. PLoS One.

[25] Desotell NF (2019). Yale University Spatiotemporal investigation of opioid-involved fatalities in Connecticut, 2009–2017.

[26] Dwyer-Lindgren L, Bertozzi-Villa A, Stubbs RW, Morozoff C, Kutz MJ, Huynh C (2016). US county-level trends in mortality rates for major causes of death, 1980–2014. JAMA.

[27] Kedir NT, Grigsby-Toussaint DS (2017). Spatial spillover and the socio-ecological determinants of diabetes-related mortality across US counties. Appl Geogr.

[28] Zhou M, Astell-Burt T, Yin P, Feng X, Page A, Liu Y (2015). Spatiotemporal variation in diabetes mortality in China: multilevel evidence from 2006 and 2012. BMC Public Health.

[29] United States Census Bureau (2016). TIGER/Line shapefiles.

[30] Centers for Disease Control and Prevention (2004). Instructions for completing the cause-of-death section of the death certificate.

[31] World Health Organization ICD-10 version 2016.

[32] State of Michigan (2015). Search for school districts.

[33] United States Census Bureau American factfinder-search.

[34] ESRI (2015). ArcGIS desktop.

[35] Klein RJ, Schoenborn CA (2001). Age adjustment using the 2000 projected U.S. population. Healthy People 2010 Stat Notes.

[36] Centers for Disease Control and Prevention (2015). Underlying 1999–2014.

[37] Anselin L (2003). GeoDa^™^ 0.9 user’s guide.

[38] Anselin L, Syabri I, Kho Y (2006). GeoDa: an introduction to spatial data analysis. Geogr Anal.

[39] Esri (2016). How create space time cube works.

[40] Interstate Technology & Regulatory Council (ITRC) 5,5 Trend test.

[41] Esri (2016). How emerging hot spot analysis works.

[42] Kochanek KD, Murphy SL, Xu J, Tejada-Vera B (2016). Deaths: final data for 2014. Natl Vital Stat Rep.

[43] Tobler WR, Gale S, Olsson G (1979). Cellular geography. Philosophy in geography.

[44] Shrestha SS, Kirtland KA, Thompson TJ, Barker L, Gregg EW, Geiss L (2012). Spatial clusters of county-level diagnosed diabetes and associated risk factors in the United States. Open Diabetes J.

[45] Laraia BA, Blanchard SD, Karter AJ, Jones-Smith JC, Warton M, Kersten E (2014). Spatial pattern of body mass index among adults in the diabetes study of Northern California (DISTANCE). Int J Health Geogr.

[46] Openshaw S (1977). A geographical solution to scale and aggregation problems in region-building, a geographical solution to scale and aggregation problems in region-building, partitioning and spatial modelling. Trans Inst Br Geogr.

[47] Manley D, Flowerdew R, Steel D (2006). Scales, levels and processes: studying spatial patterns of British census variables. Comput Environ Urban Syst.

[48] Matisziw TC, Grubesic TH, Wei H (2008). Downscaling spatial structure for the analysis of epidemiological data. Comput Environ Urban Syst.

